# Protective Effect of Tyrosol and *S*-Adenosylmethionine against Ethanol-Induced Oxidative Stress of Hepg2 Cells Involves Sirtuin 1, P53 and Erk1/2 Signaling

**DOI:** 10.3390/ijms17050622

**Published:** 2016-04-26

**Authors:** Paola Stiuso, Maria Libera Bagarolo, Concetta Paola Ilisso, Daniela Vanacore, Elisa Martino, Michele Caraglia, Marina Porcelli, Giovanna Cacciapuoti

**Affiliations:** Department of Biochemistry, Biophysics, and General Pathology, Second University of Naples, Via L. De Crecchio 7, 80138 Naples, Italy; marialibera.bagarolo@unina2.it (M.L.B.); concettapaola.Ilisso@unina2.it (C.P.I.); daniela.vanacore@unina2.it (D.V.); elisa.martino@unina2.it (E.M.); michele.caraglia@unina2.it (M.C.); marina.porcelli@unina2.it (M.P.); giovanna.cacciapuoti@unina2.it (G.C.)

**Keywords:** Tyrosol, *S*-adenosylmethionine, Sirtuin 1, Hepg2, ethanol

## Abstract

Oxidative stress plays a major role in ethanol-induced liver damage, and agents with antioxidant properties are promising as therapeutic opportunities in alcoholic liver disease. In the present work, we investigated the effect of *S*-adenosylmethionine (AdoMet), Tyrosol (Tyr), and their combination on HepG2 cells exposed to ethanol exploring the potential molecular mechanisms. We exposed HepG2 cells to 1 M ethanol for 4 and 48 h; thereafter, we recorded a decreased cell viability, increase of intracellular reactive oxygen species (ROS) and lipid accumulation, and the release into culture medium of markers of liver disease such as triacylglycerol, cholesterol, transaminases, albumin, ferritin, and homocysteine. On the other hand, AdoMet and Tyrosol were able to attenuate or antagonize these adverse changes induced by acute exposure to ethanol. The protective effects were paralleled by increased Sirtuin 1 protein expression and nuclear translocation and increased ERK1/2 phosphorylation that were both responsible for the protection of cells from apoptosis. Moreover, AdoMet increased p53 and p21 expression, while Tyrosol reduced p21 expression and enhanced the expression of uncleaved caspase 3 and 9, suggesting that its protective effect may be related to the inhibition of the apoptotic machinery. Altogether, our data show that AdoMet and Tyrosol exert beneficial effects in ethanol-induced oxidative stress in HepG2 cells and provide a rationale for their potential use in combination in the prevention of ethanol-induced liver damage.

## 1. Introduction

Chronic alcohol consumption is identified as a major risk factor for the development of liver disease. Alcoholic liver disease is characterized by a pathological process with progressive liver damage leading to steatosis, steatohepatitis, fibrosis and finally cirrosis [1]. Recent studies have pointed to a significant role for epigenetic mechanisms in the development and progression of liver disease [2]. These epigenetic effects are mainly attributable to metabolic stress generated by ethanol metabolism [3,4]. In patients with alcoholic liver disease, oxidative ethanol metabolism results in the production of compounds such as acetaldehyde and acetate that, similarly to ethanol itself, increase histone-3 acetylation [2,5]. In addition, acetaldehyde is a highly reactive compound and is toxic for hepatocytes. Its metabolism causes increased NADH/NAD^+^ ratio in the cytoplasm and mitochondria that results in inhibition of mitochondrial β-oxidation and accumulation of intracellular lipids leading to steatosis [6,7]. Acetaldehyde also promotes lipid peroxidation and mitochondrial damage [8,9] and exerts a direct inhibitory effect on enzymes implicated in DNA and histone methylation [10,11]. The microsomal electron transport system also oxidizes ethanol via catalysis by the cytochrome P450 enzymes. The 2E1 isoform of the cytochrome P450 system is induced during chronic alcohol consumption and results in enhanced production of reactive oxygen species (ROS) in the liver [12] that have been shown to play a role in ethanol-induced histone acetylation [13]. Finally, a redox-sensitive class III histone deacetylase molecule, Sirtuin 1 (SIRT1), is decreased in alcohol-exposed rat hepatocytes and in livers of alcohol-fed rats [14].

An additional important consequence of chronic alcohol consumption is the dysregulation of methionine metabolism. In fact, ethanol has been reported to inhibit the activity of a vital cellular enzyme, methionine synthase [11], leading to a significant reduction in the synthesis of *S*-adenosylmethionine (AdoMet), a crucial methyl donor for DNA and histone methylation and a key metabolite that regulates hepatocyte growth, differentiation and death. In the liver, AdoMet, through its conversion to cysteine via the trans-sulfuration pathway, also plays an important role in regulating the levels of glutathione (GSH), the well-known antioxidant molecule involved in the prevention of liver injury. The impaired homocysteine (Hcy) remethylation by methionine synthase causes increased Hcy levels both in plasma [15] and in hepatocytes [16]. It should be stressed that that elevated Hcy levels can contribute to the development of histopathological damage in the liver through induction of oxidative liver injury and endoplasmic reticulum stress. Adomet has been previously used to protect hepatic cells from ethanol insult—for example in: (a) *S*-adenosyl-l-methionine attenuates oxidative stress and hepatic stellate cell activation in an ethanol-lipopolysaccharide LPS-induced fibrotic rat model [17]; and (b) *S*-adenosyl-methionine decreases ethanol-induced apoptosis in primary hepatocyte cultures by a c-Jun N-terminal kinase activity-independent mechanism [18].

Several studies have demonstrated that many plant-derived phytochemical compounds exert their protective effects through mechanisms not strictly related to their scavenging properties. Tyrosol has been reported to reduce oxidative stress in several types of cells—for example in: (a) olive oil and its phenolic constituent Tyrosol attenuates dioxin-induced toxicity in peripheral blood mononuclear cells via an antioxidant-dependent mechanism [19]; (b) protective effect of hydroxytyrosol and Tyrosol against oxidative stress in kidney cells [20]; (c) Tyrosol attenuates in diet-induced obese mice hepatic oxidative stress [21]. Data from our laboratory have shown that Tyrosol (Tyr), a weak antioxidant monophenolic compound, specifically counteracts Intercellular Adhesion Molecule 1 (ICAM-1) increased expression induced by Hcy in EA.hy 926 cells. Interestingly, in the same experimental conditions, Tyrosol is not able to antagonize the increased expression of ICAM-1 induced by tumor necrosis factor α, allowing for hypothesizing that Tyrosol could affect specifically the Hcy-activated signaling through redox-independent mechanisms that remain to be elucidated [22]. Combinations with AdoMet of other antioxidants have also been used—for example, in hepatoprotective effects of *S*-adenosylmethionine and silybin on canine hepatocytes *in vitro* [23].

In the present study, the protective effect of AdoMet, Tyrosol, and of their combination on HepG2 human hepatocellular carcinoma cells subjected to short-term acute ethanol exposure has been investigated and the potential underlying molecular mechanisms have been explored.

## 2. Results

### 2.1. Tyrosol and AdoMet Affect Lipid Peroxidation and Fat Accumulation in HepG2 Cells Exposed to Acute Ethanol Treatment

The hepatotoxic effect of ethanol directly correlates with its exposure time and concentrations [24,25,26]. High concentrations of ethanol lead to necrotic cell death [27] while low ethanol concentration preferentially causes apoptosis [28]. To investigate the protective effect of AdoMet and Tyrosol on acute ethanol-induced cellular toxicity, we utilized HepG2 cells, a cell model that metabolizes ethanol through ADH4 [29,30].

**Figure 1:**
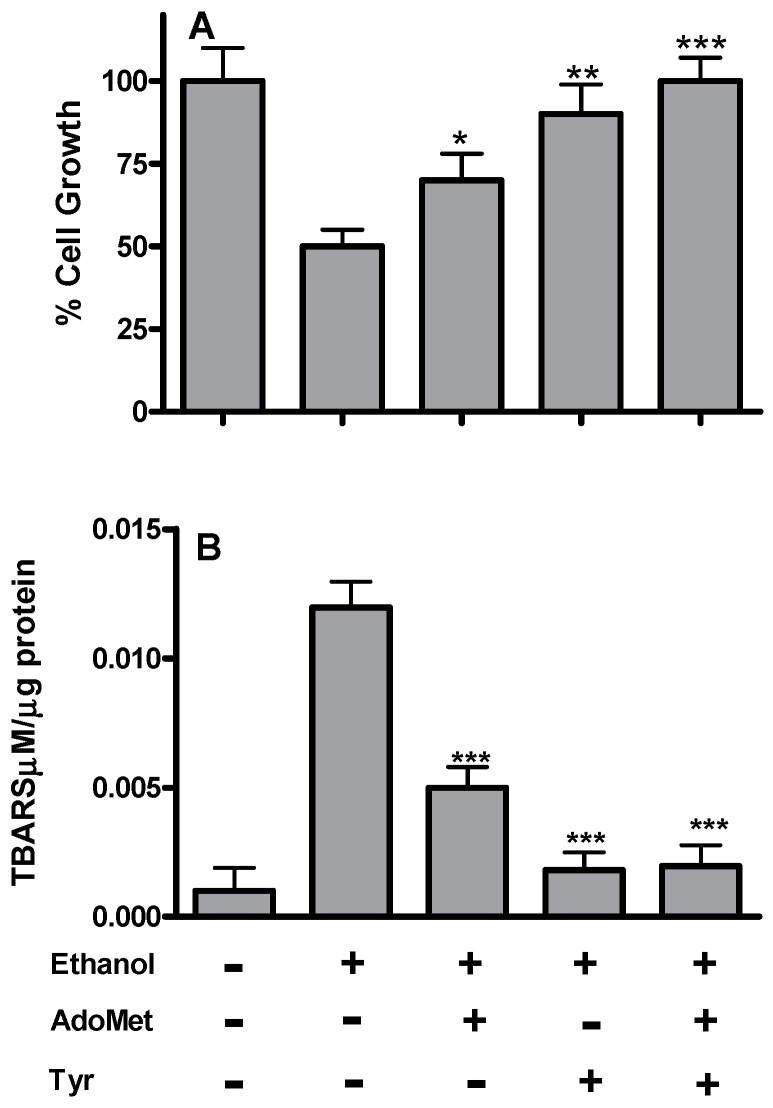
Effects of AdoMet and Tyr on cell viability and TBARS content of HepG2 cells after acute ethanol treatment. Cells were pretreated with 1 M ethanol for 4 h (Et-HepG2) and then incubated for 48 h with AdoMet 100 µM, Tyr 10 µM, and their combination. (**A**) cell proliferation was evaluated by MTT assay and was expressed as percent of Et-HepG2 cells; (**B**) TBARS content was assessed from HepG2 cell lysates. Values represent the mean ± SD of three experiments. * *p* < 0.05; ** *p* < 0.01; *** *p* < 0.001 was considered significant for ethanol alone.

The toxicity of acute ethanol treatment was assessed after 48 h by measuring the cell viability of the HepG2 cells after being pre-treated with 0.125–1 M ethanol for 1, 2, 3 and 4 h by MTT assay. The cells appeared to be quite resistant to ethanol up to 0.5 M but 1.0 M ethanol resulted in a severe loss of cell viability. Based on these results, the half maximal inhibitory concentration (IC_50_) value for cell viability was assumed to be 1.0 M ethanol for 4 h (data not shown). All the following experiments were carried out on 1 M ethanol-pretreated HepG2 cells (Et-HepG2) and after 48 h incubation with or without AdoMet, Tyrosol, and their combination. The incubation of HepG2 cells with 1 M ethanol for 4 h induced (Figure 1A) about 50% growth inhibition compared to the untreated controls (HepG2 without ethanol treatment). The addition of AdoMet or Tyrosol to the ethanol-pretreated cells exerted a protective effect reducing cell growth inhibition to about 25% and 10%, respectively. Moreover, AdoMet/Tyrosol combination completely antagonized ethanol-induced cell growth inhibition, suggesting a significative additive effect between AdoMet and Tyrosol in reducing ethanol-induced cytotoxicity.

Moreover, ethanol exposure induces oxidative stress that results in decreased GSH levels and enhanced peroxidation of lipids, proteins, and DNA [31].

The effect of AdoMet and Tyrosol on ethanol-induced lipid peroxidation was assessed by measuring the extent of lipid degradation products such as, malondyaldehyde and other aldehydes reactive to thiobarbituric acid. As shown in Figure 1B, ethanol significantly (*p* < 0.0001) increased TBARS cell content of about 12-fold more than untreated control cells. Cell treatment with AdoMet and Tyrosol determined a significant protection against lipid peroxidation but at a different extent. In fact, AdoMet treatment reduced ethanol-induced TBARS to about 2.4-fold more than untreated controls while both Tyrosol and the combination completely antagonized the TBARS increase induced by ethanol.

To additionally explore the protective effect of AdoMet and Tyrosol against acute ethanol-induced cytotoxicity, we evaluated HepG2 cell release into the culture medium of some markers reported to be correlated to alcoholic liver diseases, such as transaminases, albumin, ferritin, and neutral lipids. Moreover, we evaluated the levels of homocysteine (Hcy) as impaired Hcy trans-sulfuration has been recently considered as an indicator of alcoholic liver disease [20]. As shown in Table 1, 4 h cell exposure to 1 M ethanol induced after 48 h incubation a statistically significant increase of AST and neutral lipids, such as triacylglycerol (TG) and cholesterol (CHO). In addition, ferritin, albumin, and Hcy concentrations were all significantly higher than those observed in untreated control cells (*p <* 0.0042 for all comparisons) (Table 1). As shown in Table 1, AdoMet and Tyrosol antagonized or partially inhibited the ethanol-induced detrimental effects (Table 1). Interestingly, AdoMet specifically reduced Hcy levels (by a factor of 2), as expected. The AdoMet/Tyrosol combination was able to significantly reduce all the effects induced by ethanol with the exception of TG.

Enhanced hepatic levels of cytochrome P4502E1 (CYP2E1) represent a significant source of superoxide anion radical [32]. The acute ethanol exposure of HepG2 cells that do not express CYP2E1 induced only little changes of mithocondrial superoxide anions (MSAs) level, while fat accumulation increased about two-fold if compared to untreated controls, as demonstrated by ORO-staining (Table 2). Following AdoMet treatment, MSA production increased about 27% with a concomitant significant reduction of lipid accumulation. AdoMet could induce the lipid metabolism that consequently increases mitochondrial superoxide anions, while Tyrosol Et-HepG2 treatment produces a slight decrease in both mitochondrial superoxide anions and neutral lipid accumulation. The combination AdoMet/Tyrosol reduces both accumulation of neutral lipids and mitochondrial superoxide anions. The protective effect of AdoMet and Tyrosol appears to involve lipid homeostasis and protection against ROS generation, respectively.

### 2.2. Tyrosol and AdoMet Affect SIRT1 Expression and Antagonize Ethanol-Induced SIRT1 Nucleo-Cytoplasmic Shuttling

**Figure 2:**
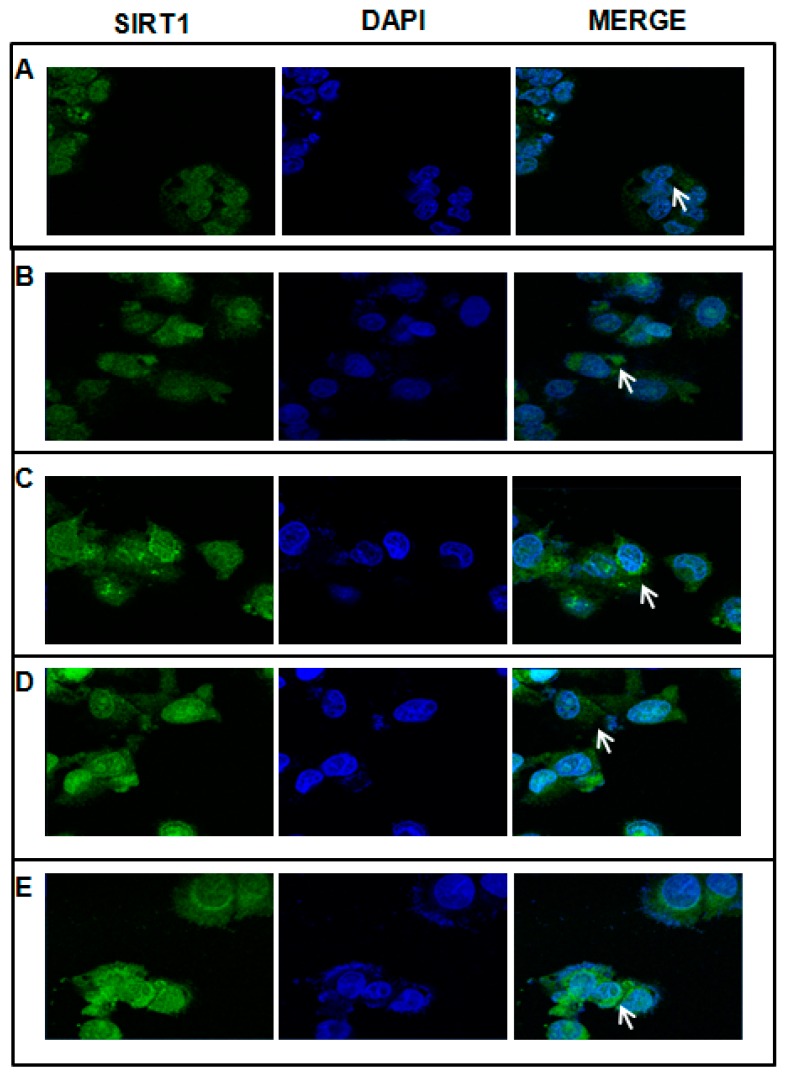
Effect of AdoMet and Tyr on subcellular localization of SIRT1. HepG2 without treatment (**A**); Et-HepG2 (cells treated for 4 h with 1 M ethanol and then incubated for 48 h without ethanol) (**B**); Et-HepG2 treated for 48 h with 100 µM AdoMet (**C**); Et-HepG2 treated for 48 h with 10 µM Tyr (**D**); Et-HepG2 treated for 48 h with 100 µM AdoMet/10 µM Tyr (**E**). Cells were incubated with anti-SIRT1 (green). The nuclei were stained with DAPI (blue). The white arrows indicate the SIRT1localization. Images were obtained by an LSM-410 Zeiss confocal microscope.

**Figure 3:**
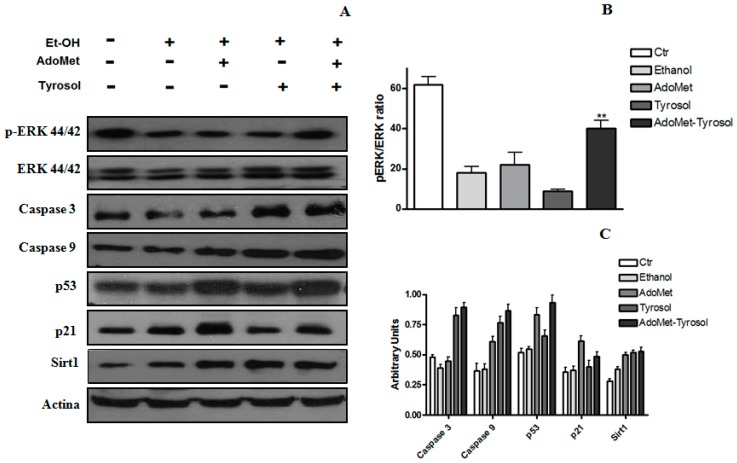
Effect of AdoMet, Tyr, and their combination on protein expression in HepG2 cells after acute ethanol treatment. The expression in cell lysates of SIRT1, Erk, p-Erk, caspase 3, caspase 9, p53, p21, and SIRT1 was assessed by Western-blot analysis using β-actin as an internal control (**A**); Densitometric quantitation of pERK/ERK ratio in different conditions (**B**); Densitometric quantification of SIRT1, caspase 3, caspase 9, p53, and p21 (**C**). Values represent the mean ± SD of three experiments. ** *p* < 0.01 was considered significant for ethanol alone.

We investigated by confocal microscopy and Western blotting whether SIRT1 expression and localization was altered during acute-ethanol-induced HepG2 cell damage. As shown in Figure 2B, after 4 h exposure to 1 M ethanol, HepG2 cells showed evenly distributed cytoplasmic immunostaining for SIRT1 but no nuclear staining, and the protein expression was at the same time increased compared to the HepG2 without ethanol treatment (Figure 3). However, the treatment with 100 μM AdoMet (Figure 2C), 10 μM Tyrosol (Figure 2D), and the combination (Figure 2E) induced a strong perinuclear and nuclear localization of SIRT1, its expression at the same time assessed by Western blot does not change compared to Et-HepG2 (Figure 3). Interestingly, a pre-treatment with the antioxidant *N*-acetylcystine largely prevented the ethanol-induced nucleocytoplasmic shuttling of SIRT1 in hepatic cells [33] indicating that SIRT1 translocation is likely a redox-dependent process. On these bases, AdoMet and Tyrosol effects on SIRT1 intracellular localization could be due to their antioxidant properties.

### 2.3. AdoMet/Tyrosol Combination Activates Proliferative Pathways

Modulation of the extracellular signal-regulated kinases ERK1/2, a signaling pathway directly associated with cell proliferation, survival, and homeostasis, has been implicated in several sicknesses including alcoholic liver disease [34,35]. As indicated by the Western blot analysis reported in Figure 3A, when compared to HepG2 cells after exposure to acute ethanol, the treatment for 48 h with AdoMet/Tyrosol combination induced an increase of ERK1/2 phosphorylated isoforms [36]. On the bases of these reports, our results suggest that the modulation of ERK1/2 signaling pathway could represent one of the underlying molecular mechanisms of the protective effects exerted by AdoMet/Tyrosol combination against acute ethanol-induced HepG2 citotoxicity.

The tumor suppressor p53 is a transcription factor that regulates a broad range of processes among which cell cycle arrest, senescence, and apoptosis are the best characterized. It has been demonstrated that the activation of p53 is needed for ethanol-induced apoptosis [37] and that the genetic elimination of p53 removes ethanol-induced liver damage [38]. We investigated the effects of AdoMet and Tyrosol on the expression of p53 and p21 and on the expression of apoptosis-related factors such as, caspase 3 and caspase 9 in HepG2 cells after acute ethanol treatment. As shown in Figure 3, AdoMet alone or in combination with Tyrosol induced an increase of the expression of p53 and, even more, of p21 whose elevated levels are able to mediate p53-induced growth arrest in response to DNA damage. On the other hand, the exposure of cells to Tyrosol decreased p21 expression and enhanced the expression of uncleaved caspase 3 and 9 suggesting that the protective effect of Tyrosol against acute ethanol-induced HepG2 damage may be related to the inhibition of cell apoptosis.

## 3. Discussion

In this study, we focused on the possible mechanism by which AdoMet and Tyrosol exert their protective effect on the oxidative damage induced by acute ethanol treatment of HepG2 cells, a cell model that does not express class I ADH genes or cytochrome P450 2E1, but metabolize ethanol through ADH4 [29,30]. The human alcohol dehydrogenase (ADH) family of proteins, comprising seven isozymes expressed as a cluster on chromosome 4q23, catalyzes the reversible oxidation of a wide range of alcohols. The class I isozymes ADH1A, ADH1B and ADH1C account for approximately 70% of alcohol metabolism in the liver, with ADH4 contributing for most of the remaining 30% [30]. Ethanol oxidation by ADH and the subsequent metabolism of acetaldehyde product dramatically alter NADH/NAD^+^ ratio, and, as a consequence, the intracellular redox state, causing inhibition of mitochondrial β-oxidation and accumulation of intracellular lipids and finally leading to steatosis [6,7].

Enhanced hepatic levels of cytochrome P4502E1 (CYP2E1) may play a key role in the pathogenesis of some liver diseases as CYP2E1 represents a significant source of ROS, such as superoxide anion radical [32]. The results of the effect of AdoMet and Tyrosol on mitochondrial superoxide anions and lipid accumulation, obtained by ORO staining, are in agreement with recent reports suggesting that methyl-donor administration to animals fed with a high fat diet increases the activity of hepatic AMP-activated protein kinase (AMPK) and increases fatty acid oxidation contributing to reduced hepatic lipid accumulation [31]. It is conceivable that the increase of mitochondrial superoxide anions (MSA) in AdoMet-treated HepG2 cells may be due to increased fatty acids β-oxidation, a process that can lead to the generation of high intracellular ROS concentrations including superoxide, hydrogen peroxide, and hydroxyl radicals, responsible for an inflammatory status associated with cellular injury. SIRT1, a NAD^+^-dependent class III histone deacetylase, is involved in the regulation of many physiological functions such as longevity, apoptosis and oxidative stress improving hepatic and acute liver injury.

**Figure 4:**
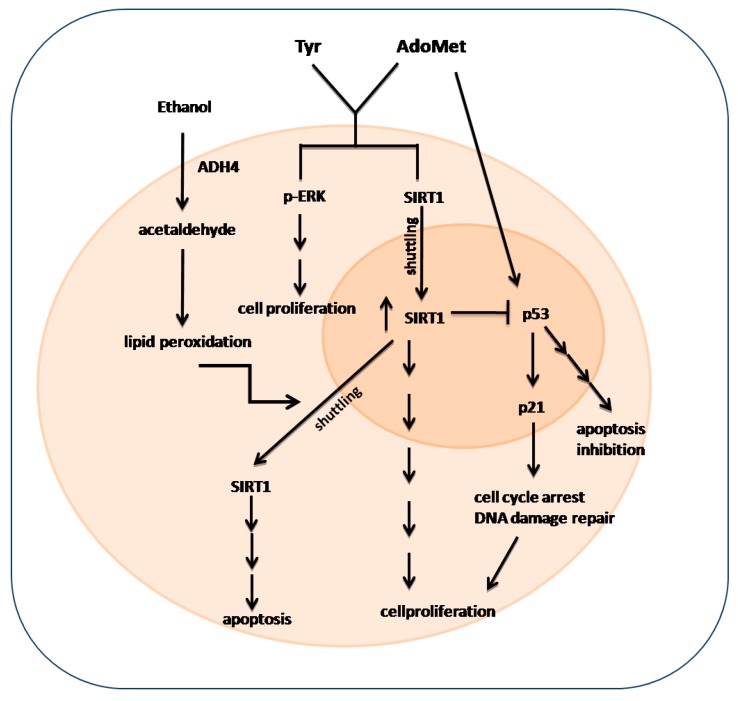
The addition of Tyr and AdoMet to hepatocellular carcinoma cells after acute ethanol treatment increases pERK expression that, in turn, determines increased cell proliferation. The treatment also induces an increase of nuclear Sirt1 expression that both promotes cell proliferation itself and blocks the p53/p21 pathway with consequent apoptosis inhibition.

SIRT1 plays an important role in alcoholic liver disease. SIRT1 is predominately placed in the nucleus where its activity induces several transcriptional responses through the deacetylation of a large range of transcriptional regulators. A number of stimuli can lead to the translocation of SIRT1 from the nucleus to the cytoplasm, subsequently impairing SIRT1 activity. In Figure 4, the speculative scheme of Sirtuin 1, P53 and Erk1/2 signaling activated by ethanol Hepg2 treatment was reported. The cytotoxic effect of acute ethanol treatment leads to an increased lipid peroxidation, which, in turn, induces a partial relocalization of SIRT1 into the cytoplasm where it enhances apoptosis through a mechanism independent of its deacetylase activity [39,40]. In fact, the ethanol-mediated disruption of SIRT1 signaling leads to excessive fat accumulation and to inflammatory responses in animal and human livers [41]. It has been reported that exposure to ethanol can induce a transport of SIRT1 from the nucleus to the cytoplasm in either cultured hepatocytes or in animal livers [41,42,43]. Moreover, SIRT1 activation inhibits NF-κB signaling and enhances oxidative metabolism and the resolution of inflammation. Three considerations can be deduced from the results obtained: (i) AdoMet affects p53-p21 signaling. As shown in Figure 3, a significant increase in the expression of p53 and p21 is observable after the treatment of cells for 48 h with 100 μM AdoMet. p53 can be regulated by upstream signaling pathways in response to cellular stresses in many different ways. It is well known that p53 plays important but context-dependent roles in cellular responses to low or high levels of oxidative stress. In response to low levels of oxidative stress, p53 exhibits antioxidant activities and assists the survival and repair of cells with minor injuries while in response to high levels of oxidative stress, p53 exhibits prooxidative activities and induces cellular apoptosis [44]. In the liver, AdoMet is a precursor of GSH, the major endogenous antioxidant that protects cells against injury by scavenging free radicals involved in the pathogenesis of alcoholic liver disease [21]. The sulfonium compound, therefore, could provide protection by facilitating the antioxidant activities of p53. The increase of p21 expression subsequent to the increase of p53 will lead to cell arrest until the damage is sufficiently repaired; (ii) Tyrosol causes an evident decrease of p21 expression and an increase of uncleaved caspase 9 and 3, suggesting that this compound not only plays an important role as an antioxidant molecule but also possesses the ability to modulate the expression of genes that regulates apoptosis; and (iii) the protective effect exerted by AdoMet/Tyrosol combination is associated with an increase of ERK1/2 phosphorylation and with increased SIRT 1 protein and its nuclear relocalization. In the nucleus, Sirt1 interacts, deacetylates, and thereby negatively regulates the transactivation function of several key transcription factors, such as p53, thus playing a central role in the determination of the cellular fate (apoptosis/survival).

ERK1/2-regulated pathways play an important role in controlling lipid metabolism in the liver. It has been reported that ethanol-induced abnormal hepatic methionine metabolism is specifically associated with the suppression of transmethylation reactions and with the inhibition of MEK/ERK1/2 activation, and that betaine supplementation, improving the AdoMet/AdoHcy ratio, is able to alleviate ERK1/2 activity inhibition [45]. Moreover, it has also been described that the polyphenols, fisetin and resveratrol, provide neuroprotection in multiple models of Huntington’s disease due to their ability to activate ERK [46].

Sirt1 up-regulation, therefore, appears to be a common step among the crossroads of the signaling pathways affected by AdoMet and Tyrosol, responsible for the switch of cells from apoptosis, caused by ethanol-induced oxidative stress, to proliferation.

In conclusion, our data show that AdoMet/Tyrosol combination is beneficial in acute ethanol-induced HepG2 cell injury and strongly suggest a potential use of these molecules in combination to support liver function altered by ethanol damage.

## 4. Material and Methods

### 4.1. Chemicals

RPMI 1640, bovine serum albumin (BSA) and 3-(4,5-dimethylthiazol-2-yl)-2,5-diphenyl tetrazolium bromide (MTT) were purchased from Sigma-Aldrich (St. Louis, MO, USA). Phosphate-buffered saline (PBS) and trypsin-EDTA were from Lonza (Milano, Italy). Fetal bovine serum (FBS) was purchased from Gibco (Grand Island, NY, USA) and Tyrosol (*p*-hydroxyphenylethanol, Tyr) from Sigma-Aldrich. AdoMet was provided from New England Biolabs (Hitchin, UK), prepared in a solution of 0.005 M H_2_SO_4_ and 10% ethanol, filtered and stored at −20 °C. Western blot analysis was performed using the following primary antibodies: monoclonal antibody (mAb) raised against caspase 3 was from Enzo Life Sciences (Florence, Italy); mAb raised against caspase 9, polyclonal antibody (polyAb) p-ERK 44/42 and polyAb ERK 44/42 from Cell Signaling Technology (Beverly, MA, USA); mAb actin, mAb p53 and polyAb p21 were from Santa Cruz Biotechnology (San Diego, CA, USA). Blots were incubated with horseradish peroxidase (HRP)-conjugated goat anti-rabbit or HRP-conjugated goat anti-mouse (Immunoreagents Inc., Raleigh, NC, USA) secondary antibodies. Anti-SIRT1 mAb was purchased from Santa Cruz Biotechnology and secondary antibodies conjugated to Alexafluor 488 from Life Science, Portland, OR, USA and 4′,6-Diamidino-2-phenylindole (DAPI) from Sigma-Aldrich. All buffers and solutions were prepared with ultra-high quality water. All reagents were of the purest commercial grade.

### 4.2. Cell Culture

Human hepatocellular carcinoma cells (HepG2, HB-8065) were obtained from the American Type Culture Collection (ATCC, Manassas, VA, USA) and cultured in RPMI 1640 supplemented with 10% heat-inactivated FBS, 100 U/mL penicillin, 100 μg/mL streptomycin, and 1% l-glutamine. The cells were grown in a humidified atmosphere of 95% air/5% CO_2_ at 37 °C.

### 4.3. Cell Viability

HepG2 cells were seeded in 96-well plates at the density of 5 × 10^3^ cells/well in RPMI complete medium. After 4 h incubation at 37 °C, the cells were pre-treated with 1.0 M ethanol for 4 h following by treatments with 100 μM AdoMet and 10 μM Tyrosol, alone or in combination for 48 h. Cell viability was assessed by adding 3-(4,5-dimethylthiazol-2-yl)-2,5-diphenyl tetrazolium bromide (MTT) solution in PBS to a final concentration of 5 mg/mL as previously described [24]. The absorbance at 570 nm were measured by Bio-Rad 550 microplate reader (Bio-Rad Laboratories, Milan, Italy). Cell viability values are expressed as percentage of the control (100%). All experiments were performed in triplicate.

### 4.4. Oil Red O Staining

Oil Red O (ORO) staining was performed as previously described [47]. The fixed cells (10% formalin solution) were incubated with isopropanol (60%), and stained for 10 min with ORO solution. Following staining, the cultures were washed and the dye was extracted by isopropanol and measured colorimetrically at 520 nm. Results were expressed as A_520_/cells number.

### 4.5. Western Blot Analysis

The effect of AdoMet, Tyrosol, and their combination on the expression of SIRT1, p-ERK, ERK, p53 and p21 was determined by Western blot analysis. For the preparation of cell extracts, 2 × 10^6^ HepG2 cells were seeded in tissue culture dishes and incubated with and without AdoMet, Tyrosol and AdoMet/Tyrosol combination. The cells were lysed and the proteins were extract as previously described [48]. Equal amounts of cell proteins were resolved on (SDS)-polyacrylamide gels and transferred to nitrocellulose membrane by Trans blot turbo (Bio-Rad, Hercules, CA USA). For immunodetection, membranes were incubated O/N with SIRT1, p-ERK, ERK, p53 and p21 antibodies as recommended by the manufacturer. This step was followed by incubation with corresponding horseradish peroxidase (HRP)-conjugated secondary antibody. Protein bands were detected by chemiluminescence detection reagents (Millipore Corporation, Darmstadt, Germany). The protein bands were quantified using the NIH Image J system (National Institute of Health, Bethesda, MD, USA).

### 4.6. Mitochondrial Superoxide Anion Levels

Mitochondrial superoxide anions (MSAs) levels were determined by hydroethidine (HE) staining by FACScan flow cytometer (FACScan, BD Biosciences UK) as previously described [24]. For each sample, 2 × 10^4^ events were acquired. Analysis was carried out in triplicate in at least three separate experiments.

### 4.7. Thiobarbituric Acid-Reactive Species (TBARS) Levels

Samples were incubated with a solution consisting of 0.5 mL of 20% acetic acid, and 0.5 mL of 0.78% aqueous solution of thiobarbituric acid (pH 3.5), heating at 95 °C for 45 min, and than centrifuged at 4000 rpm for 5 min. The aldheide reactive at thiobarbituric acid (TBARS) were quantified at 532 nm [49]. Results were expressed as TBARS µM/µg of protein. Each data point is the average of triplicate measurements with each individual experiment performed in duplicate.

### 4.8. Laboratory Parameters

Aspartate transaminase (AST) activity, alanine transaminase (ALT) activity, total cholesterol (CHO), triglycerides (TG), ferritin, Hcy, and γ-glutamyltransferase (G-GT) were determined using standard clinical chemical methods.

### 4.9. Immunostaining and Confocal Microscopy

HepG2 cells growing in 24-well plates at the density of 10 × 10^3^ cells/well were treated as described above, fixed using PBS 4% paraformaldehyde for 10 min and then permeabilized for 10 min with PBS 0.1% Triton X-100. Immunostaining was carried out by O/N incubation at 37 °C with specific antibodies against SIRT1 (1:1000; Sigma Aldrich). Cells were then incubated with secondary antibodies conjugated to Alexafluor 488 (1:1000) for 1 h at room temperature. Cells were stained for 2 min at room temperature by 4′,6-diamidino-2-phenylindole, dihydrochloride (DAPI, 5 µ/mL), a nuclear and chromosome counterstain that emits blue fluorescence upon binding to AT regions of DNA. The cells were analyzed by an LSM-410 Zeiss confocal microscope (Carl Zeiss Microscopy Ltd., Cambridge, UK).

### 4.10. Statistical Analysis

All experiments were performed three times with replicate sample. Data are expressed as mean ± SD (standard deviation). The means were compared using analysis of variance (ANOVA) plus Bonferroni’s *t*-test. A *p*-value of <0.05 indicates a statistically significant result.

## Figures and Tables

**Table 1:** Evaluation of liver enzyme, protein level and neutral lipid into the culture medium of EtOH-HepG2 treated for 48 h with AdoMet, Tyrosol and AdoMet-Tyrosol combination.

	HepG2	Et-HepG2	Et-HepG2 + AdoMet	Et-HepG2 + Tyr	Et-HepG2 + AdoMet-Tyr
AST/GOT	0.10 ± 0.01	0.14 ± 0.02	0.12 ± 0.015	0.14 ± 0.02	0.11 ± 0.01
ALT/GOT	0.027 ± 0.003	0.031 ± 0.002	0.034 ± 0.003	0.050 ± 0.001	0.029 ± 0.002
CHO	0.34 ± 0.03	0.77 ± 0.015	0.37 ± 0.03	0.54 ± 0.04	0.34 ± 0.05
TG	0.44 ± 0.05	1.0 ± 0.21	0.88 ± 0.06	1.2 ± 0.1	0.70 ± 0.06
Albumin g/L	0.06 ± 0.03	0.35 ± 0.08	0.13 ± 0.009	0.30 ± 0.01	0.07 ± 0.003
Ferritin µg/L	0.90 ± 0.1	2.4 ± 0.3	1.4 ± 0.4	2.3 ± 0.2	1.1 ± 0.3
Homocysteine µmol/L	0.25 ± 0.06	0.56 ± 0.05	0.26 ± 0.03	0.54 ± 0.04	0.26 ± 0.037

EtOH−Hepg2 = hepg2 treated with ethanol 1 M for 4 h; All the results were reported as values per protein concentration.

**Table 2:** Effect of AdoMet, Tyr and AdoMet/Tyr combination on mitochondrial superoxide anions (MSA) and neutral lipid accumulation.

	MSAs	ORO A_520_nm/Cells
HepG2	9.3 ± 0.6	3.3 × 10^−5^ ± 0.1 × 10^−5^
Et-HepG2	9.6 ± 0.57	6.8 × 10^−5^ ± 0.25 × 10^−5^
Et-HepG2 + AdoMet	11.8 ± 0.6	5.0 × 10^−5^ ± 0.34 × 10^−5^
Et-HepG2 + Tyr	9.0 ± 0.8	6.0 × 10^−5^ ± 0.29 × 10^−5^
Et-HepG2 + AdoMet/Tyr	8.5 ± 0.5	5.3 × 10^−5^ ± 0.37 × 10^−5^

Et-HepG2 = HepG2 treated with 1 M ethanol for 4 h and then incubated without ethanol for 48 h. MSAs (mithocondrial superoxide anions) were evaluated hydroethidine (HE) staining by FACScan flow cytometer; Lipid accumulation was analyzed by ORO staining and quantified by measuring the absorbance at 520 nm.

## Abbreviations

AdoMet	*S*-Adenosylmethionine
MAPK	mitogen-activated protein kinase
ERK1/2	extracellular signal-regulated kinases 1 and 2
Tyr	Tyrosol
ROS	reactive oxygen species
Hcy	homocysteine
ADH	alcohol dehydrogenase
BSA	bovine serum albumin
MTT	3-(4,5-dimethylthiazol-2-yl)-2,5-diphenyl tetrazolium bromide
PBS	phosphate-buffered saline
FBS	fetal bovine serum
SDS	sodium dodecyl sulphate
TBARS	thiobarbituric acid-reactive species
HE	hydroethidine
AST	aspartate transaminase
ALT	alanine transaminase
G-GT	γ-glutamyltransferase
TG	triacylglycerol
CHO	total cholesterol
MSA	mithocondrial superoxide anions
ORO	Oil Red O
SIRT1	Sirtuin 1
pERK	phosphorylated ERK
HRP	horseradish peroxidase
MFIs	mean fluorescence intensity
